# Impact of hypertension and angiotensin receptor blockers on colorectal cancer: A retrospective study

**DOI:** 10.1097/MD.0000000000045372

**Published:** 2025-10-24

**Authors:** Mohammad Rezazadeh, Shahram Agah, Amirreza Kamyabi, Ramtin Ghamkhari Pisheh, Amirhossein Eshraghi, Seidamir Pasha Tabaeian, Abolfazl Akbari

**Affiliations:** a Student Research Committee, School of Medicine, Iran University of Medical Sciences, Tehran, Iran; b Colorectal Research Center, Iran University of Medical Sciences, Tehran, Iran.

**Keywords:** angiotensin receptor blockers, colorectal cancer, hypertension

## Abstract

Colorectal cancer (CRC) is a common malignancy of the gastrointestinal tract and the second leading cause of cancer-related death. Hypertension (HTN) is the primary cause of death worldwide and a significant risk factor for malignancies, leading to the new concept of “Onco-Hypertension.” Angiotensin receptor blockers (ARB) are among the first-line drugs for the treatment of HTN, and in recent years, concerns have been raised about their potential carcinogenic substances. This research intended to determine the association of HTN and ARBs with the chance of developing a clinicopathological profile of CRC. This multicenter retrospective observational study comprised 800 controls without CRC and 650 patients with CRC from 2019 to 2023. Key demographic and clinicopathological data were collected. Using univariate and multivariate logistic regression models, adjusted and unadjusted odds ratios (OR) were computed to examine the impact of HTN and ARBs on the clinicopathological features and chance of developing colorectal cancer. HTN and the chance of developing CRC were initially shown to be positively significant (OR = 1.28, confidence interval = 1.02, 1.61). After adjusting for confounding variables, the OR was no longer statistically significant. An elevated chance of developing CRC was in patients with HTN who used ARB (OR = 2.65, confidence interval = 1.47, 4.79). Among the clinicopathological features of the tumor, stage (*P* value < .001) and metastasis (*P* value = .002) have a significant association with HTN. The findings of this study did not reveal a significant association between HTN and the chance of developing CRC. However, a positive association was observed between HTN and metastasis and stage. Results identify a possible signal of an increased chance of developing CRC linked to ARBs. However, further observational and analytical studies are necessary to elucidate the underlying mechanisms and confirm these findings.

## 1. Introduction

Colorectal cancer (CRC) is a common malignancy of the gastrointestinal tract and the second leading cause of cancer-related death.^[1]^ CRC was among the most common cancers in Iran in 2016 and is predicted to remain among the leading cancers nationally in 2025 with the expected increase of cases equal to 54.1%.^[2]^ Increased body mass index, red meat intake, cigarette smoking, low physical activity, low vegetable consumption, and low fruit consumption were associated with increased risk of CRC.^[3]^ The clinicopathological features of CRC affect the prognosis and survival of the patients. The 5-year survival rate for CRC is 64%, but it decreases to 12% for metastatic CRC.^[4]^ Also, some evidence suggests that patients with a right-sided primary or poorly differentiated tumor have a worse prognosis.^[5]^ Therefore, it is essential to understand the factors affecting the clinicopathological profile of CRC.

Although many studies have been conducted in the field of understanding carcinogenesis, there isn’t a universal explanation for why it happens. One theory could be related to hypertension (HTN). Previous research has demonstrated that HTN may contribute to the development of cancer in addition to its initiation.^[6]^ HTN is a chronic illness characterized by persistently raised blood pressure in the arteries.^[7]^ The Global Burden of Disease Study 2021 estimates that high systolic blood pressure (SBP) caused 226 million disability-adjusted life years and 10.9 million deaths globally in 2021.^[8]^

Studies have noted the strong positive association between HTN and various cancers, including kidney, renal cell carcinoma, breast, colorectal, endometrial, and bladder. This close relationship has led to the new concept of “Onco-Hypertension” that has been proposed to address the mutual risks posed by HTN and cancer.^[9]^ Some studies have limited the risk to men, but others have shown that HTN is also a risk factor for cancer in women. Other risk factors, primarily smoking, obesity, and diabetes, seem to be complexly linked to HTN as a risk factor for cancer.^[10]^ While both cancer and HTN are 2 of the leading global causes of death and are expected to rise, it is important to understand the probability of comorbidity between these chronic conditions.

Considering the majority of the guidelines, including the 2017 ACC/AHA guidelines, angiotensin-converting enzyme inhibitors, calcium channel blockers, and angiotensin receptor blockers (ARBs) are among the drugs used as first-line therapy for HTN.^[11]^ Medicaid drug utilization for ACEIs and ARBs ascended by approximately 25% from 10,150,892 prescriptions in 1991 to 12,709,773 in 2021.^[12]^ ARBs are a powerful class of antihypertensive drugs that work by stopping the release of angiotensin II from the angiotensin I receptor. This results in a lowering of blood pressure by inhibiting the effects of angiotensin II on the release of catecholamines, aldosterone, arginine vasopressin, water intake, and hypertrophic response.^[13]^

There is a vast amount of literature on the antihypertensive and cardiovascular therapeutic effects of ARBs, but there are few studies on side effects, especially the association with malignancies. In 2018, the United States Food and Drug Administration regulators found that a number of ARB formulations, including N-nitrosodimethylamine, are potential human carcinogens.^[14]^ However, there is inconsistency among the results regarding the association between HTN, ARBs, and CRC. According to some studies, HTN has a key role in the development of CRC and other neoplasms.^[15]^ While other studies suggest there is no significant relationship between HTN and CRC.^[16]^ Some studies have shown that the use of ARBs inhibits tumor growth in CRC.^[17]^ This discrepancy reveals the need for additional investigations. Knowing the risk factors for CRC can help with early detection, prevention, and customized screening plans, which can eventually improve outcomes, influence lifestyle decisions, and raise awareness among high-risk groups. As a result, this study is conducted to investigate the link between the relationship between HTN and ARBs with the chance of developing and clinicopathological profile of CRC.

## 2. Methods and materials

This retrospective study was performed in 2 tertiary referral hospitals. It was approved with the ethics code IR.IUMS.REC.1402.671. This study adhered to the Strengthening the Reporting of Observational Studies in Epidemiology reporting guideline for case-control studies. The data in this research was gathered retrospectively from patients by examining medical records spanning 4 years.

### 2.1. Sample size calculation

Power and Sample software version 3.1.2 (Biostatistics, Nashville) was used to calculate the sample size for independent cases and controls with 1 control per case. At least 105 cases and controls were required for the inquiry, which had 80% study power, a 2-sided α of 0.05, and predicted proportions (prevalence of HTN) in case and control of 0.177 and 0.054 based on a comparable study by Jafari Nasab S et al.^[18]^ Based on the available sample frame, 650 cases and 800 controls were selected.

### 2.2. Study participants

The following are the inclusion criteria: patients with CRC and hospitalized patients without CRC between September 2019 and September 2023 and over 45 years old were considered as the case and control groups, respectively. The studied ARB drugs include low doses of losartan and valsartan (80 mg and 50 mg per day, respectively). In this study, HTN was defined as SBP of 130 mm Hg and/or diastolic blood pressure (DBP) of 80 mm Hg or more. Also, only cases of essential HTN were included in the study. The following are the exclusion criteria for both groups: first-order family history of cancer, having inflammatory bowel disease, or any malignant disease history. To ensure that HTN preceded CRC, only patients with HTN and ARB consumption at least 2 years before CRC diagnosis were included in the study.

### 2.3. Sampling and data collection

After applying the inclusion and exclusion criteria, 650 and 800 patients were chosen as potentially desirable cases and controls, respectively. Common characteristics collected from the case and control cohorts included age, gender, aspirin use, HTN, and ARB use. Clinicopathological features of the CRC included tumor location (proximal, distal, rectosigmoid, and overall, meaning in more than 2 of the 3 locations mentioned), tumor grade (well, moderately, and poorly differentiated), CRC stage (as defined by the PTNM AJCC 8th edition), and distant metastasis. All the information was taken from the medical documents.

### 2.4. Statistical analysis

For all statistical analyses, SPSS software version 27.0.1 (IBM, Armonk) was used. Quantitative variables were examined for normality using the Shapiro–Wilk test. The mean and standard deviation (SD) were used to represent continuous variables. Frequencies and percentages were used to describe the qualitative variables. The chi-square test was used for testing relationships between categorical variables. Univariate and multivariate binary logistic regression analysis was used to determine the factors affecting CRC development. It was also applied for clinicopathological features that were significant in chi-square analysis. 95% confidence interval (CI) and a *P*-value < .05 were used to determine statistical significance.

## 3. Results

### 3.1. Case and control cohort baseline characteristics

The descriptive statistics of both cases and controls are displayed in Table 1. The cases were 58.03 (±10.28) years old on average (±SD). This study also included 800 controls, whose mean age (±SD) was 63.27 (±9.80) years. Table 1 also shows the clinicopathological profile of colorectal tumors. The results showed that the control population has a lower proportion of patients with HTN and ARB users. The case population was younger, had a higher female proportion, and had a lower percentage of smokers. The case and control groups consumed aspirin at similar rates.

**Table 1:** Descriptive statistics and analysis of characteristics of the patients with and without CRC. The chi-square test was used for testing relationships between variables.

Variable	Patients with CRC (n = 650)	Patients without CRC (n = 800)	*P* value
Age (yr)			<.001
45–50	64 (9.9%)	234 (29.3%)	
51–55	87 (13.4%)	167 (20.9%)	
56–60	117 (18.0%)	122 (15.2%)	
61–65	128 (19.7%)	97 (12.1%)	
66–70	110 (16.9%)	85 (10.6%)	
71–75	67 (10.3%)	39 (4.9%)	
≥76	77 (11.9%)	56 (7.0%)	
Sex			
Female	261 (40.2%)	176 (22.0%)	<.001
Male	389 (59.8%)	624 (78.0%)	
Aspirin			
No	538 (82.8%)	692 (86.5%)	.057
Yes	112 (17.2%)	108 (13.5%)	
Smoking			
No	521 (80.2%)	598 (74.8%)	.016
Yes	129 (19.8%)	202 (25.2%)	
HTN			
No	443 (68.2%)	591 (73.9%)	.012
Yes	207 (31.8%)	209 (26.1%)	
ARBs			
No	538 (82.8%)	734 (91.8%)	<.001
Yes	112 (17.2%)	66 (8.2%)	
Distant metastasis			
Yes	143 (22%)	–	–
No	507 (78%)		
Grade of tumor			
Well differentiated	376 (57.8%)	–	–
Moderate differentiated	183 (28.2%)		
Poor differentiated	91 (14.0%)		
Numerical stage			
1	34 (5.2%)	–	–
2a	136 (20.9%)		
2b	79 (12.2%)		
2c	14 (2.2%)		
3a	73 (11.2%)		
3b	106 (16.3%)		
3c	64 (9.8%)		
4	144 (22.2%)		
Tumor location			
Proximal	143 (22.0%)	–	–
Distal	60 (9.2%)		
Rectosigmoid	396 (60.9%)		
Overall	51 (7.8%)		

ARB = angiotensin receptor blocker, CRC = colorectal cancer, HTN = hypertension.

### 3.2. Impact of ARBs and HTN on the chance of developing CRC

**Figure 1.**
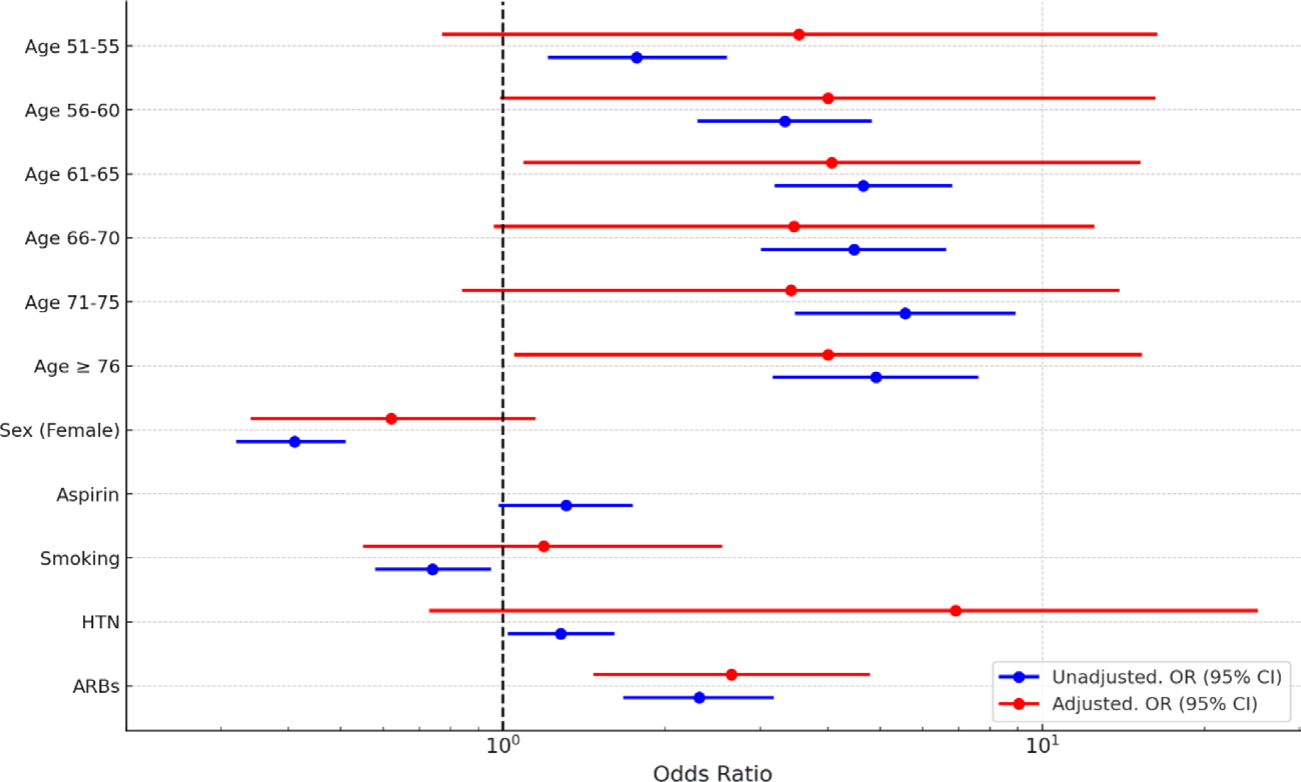
Forest plot of univariate and multivariate analysis of effect of HTN and ARBs consumption on risk of CRC. ARB = angiotensin receptor blocker, CRC = colorectal cancer, HTN = hypertension.

The impact of HTN, ARB use, and other variables on the risk of CRC is shown in Table 2 through both univariate and multivariate analysis. Results are also illustrated in Figure 1. Based on univariate analysis, all variables except aspirin use were significantly associated with CRC incidence. According to unadjusted odds ratios (OR), ARB consumers and patients with HTN have a higher chance of developing CRC (OR = 2.31, 95% CI = 1.67–3.18 and OR = 1.28, 95% CI = 1.02–1.61, respectively). After multivariate analysis, the association of all variables except ARB use became nonsignificant. Based on adjusted ORs, ARB consumers are more likely to develop CRC (OR = 2.33, 95% CI = 1.57–3.44), and HTN does not significantly affect the chance of CRC development (OR = 0.79, 95% CI = 0.59–1.05).

**Table 2:** Results of binary logistic regression to identify development of CRC predictors.

Variable	Unadjusted OR (95% CI)	*P* value	Adjusted OR (95% CI)	*P* value
Age (yr)				
45–50	Reference	–	Reference	–
51–55	1.77 (1.21, 2.60)	.003	3.54 (0.77, 16.36)	.105
56–60	3.33 (2.29, 4.83)	<.001	4.01 (0.99, 16.24)	.052
61–65	4.66 (3.19, 6.81)	<.001	4.07 (1.09, 15.23)	.037
66–70	4.48 (3.01, 6.65)	<.001	3.46 (0.96, 12.50)	.058
71–75	5.57 (3.48, 8.92)	<.001	3.42 (0.84, 13.92)	.086
≥76	4.91 (3.16, 7.62)	<.001	4.01 (1.05, 15.30)	.042
Sex				
Male	Reference	–	Reference	–
Female	0.41 (0.32, 0.51)	<.001	0.62 (0.34, 1.15)	.129
Aspirin				
No	Reference	–	–	–
Yes	1.31 (0.98, 1.74)	.065	–	–
Smoking				
No	Reference	–	Reference	–
Yes	0.74 (0.58, 0.95)	.017	1.19 (0.55, 2.55)	.659
HTN				
No	Reference	–	Reference	–
Yes	1.28 (1.02, 1.61)	.032	6.90 (0.73, 25.12)	.092
ARBs				
No	Reference	–	Reference	–
Yes	2.31 (1.67, 3.18)	<.001	2.65 (1.47, 4.79)	.001

Univariate and multivariate binary logistic regression analysis was used to determine the factors affecting CRC development. For each variable, the odds of the outcome for all categories compared to the odds of the outcome for a reference category were reported as the OR. Data are adjusted for age, aspirin (never vs ever), smoking (never vs ever), sex (female vs male), HTN (never vs ever), and ARBs (never vs ever).

ARB = angiotensin receptor blocker, CI = confidence interval, CRC = colorectal cancer, HTN = hypertension, OR = odds ratios.

### 3.3. Association of HTN and clinicopathological features of tumor

**Figure 2.**
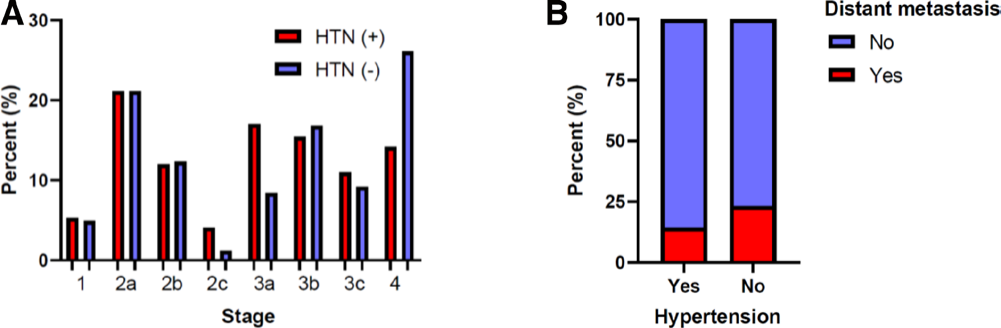
Bar chart documenting the Pearson chi squared test showing in the association between HTN is associated with tumor stage (*P* value < .001) and metastasis (*P* value = .002). Results show that patients with HTN had fewer tumors with stage 2b, 3b and 4 (A). Distant metastasis occurred less frequently in hypertensive patients (B). HTN = hypertension.

The investigation of the association of HTN and the features of tumors is presented in Table 3. In patients with and without HTN, the least common stages of CRC were 3 and 2, respectively. The most common tumor location was the rectosigmoid in both groups of patients with and without HTN. HTN has no significant association with the grade or location of tumors. However, HTN is significantly associated with the stage and metastasis of tumors (Fig. 2). A higher proportion of HTN patients have tumor metastases than patients without HTN. Table 4 shows the univariate analysis to identify metastasis and stage predictors. None of the variables were significantly associated with metastasis and tumor stage, except for aspirin use, which was significantly associated with a reduced chance of metastasis (OR = 0.54, 95% CI = 0.30–0.97).

**Table 3:** Chi-square test results in investigating the relationship between HTN and tumor clinicopathological characteristics.

Variable	Patients with HTN (n = 207)	Patients without HTN (n = 443)	*P*-value
Distant metastasis			.002
No	178 (86.0%)	334 (75.4%)	
Yes	29 (14.0%)	109 (24.6%)	
Grade of tumor			
Well differentiated	158 (76.3%)	258 (58.3%)	.456
Moderate differentiated	38 (18.4%)	130 (29.3%)	
Poor differentiated	11 (5.3%)	55 (12.4%)	
Stage			
1	11 (5.3%)	22 (5.0%)	<.001
2	77 (37.2%)	155 (35.0%)	
3	90 (43.5%)	150 (33.9%)	
4	29 (14.0%)	116 (26.2%)	
Tumor location			
Proximal	50 (24.2%)	91 (20.5%)	.436
Distal	14 (6.8%)	45 (10.2%)	
Rectosigmoid	124 (59.9%)	273 (61.6%)	
Overall	19 (9.2%)	34 (7.7%)	

**Table 4:** Results of univariate binary logistic regression to identify metastasis and stage predictors.

Variable	Metastasis		Stage	
	Unadjusted OR (95% CI)	*P* value	Unadjusted OR (95% CI)	*P* value
Age (yr)				
45–50	Reference	–	Reference	–
51–55	0.94 (0.43, 2.04)	.871	0.94 (0.47, 1.75)	.774
56–60	1.15 (0.57, 2.34)	.701	1.44 (0.77, 2.69)	.253
61–65	0.83 (0.41, 1.71)	.619	0.89 (0.49, 1.63)	.716
66–70	0.85 (0.41, 1.79)	.674	1.03 (0.55, 1.93)	.918
71–75	0.60 (0.25, 1.44)	.251	0.96 (0.48, 1.90)	.897
≥76	0.69 (0.30, 1.58)	.377	0.87 (0.45, 1.70)	.689
Sex				
Male	Reference	–	Reference	–
Female	1.13 (0.76, 1.67)	.540	1.00 (0.73, 1.37)	.997
Aspirin				
No	Reference	–	Reference	–
Yes	0.54 (0.30, 0.97)	.039	0.90 (0.59, 1.35)	.597
Smoking				
No	Reference	–	Reference	–
Yes	1.13 (0.71, 1.80)	.608	1.00 (0.68, 1.48)	.985
HTN				
No	Reference	–	Reference	–
Yes	0.87 (0.57, 1.33)	.544	0.85 (0.61, 1.19)	.343
ARBs				
No	Reference	–	Reference	–
Yes	0.68 (0.39, 1.17)	.159	0.73 (0.48, 1.09)	.124

Univariate and multivariate binary logistic regression analysis was used to determine the factors affecting tumor metastasis and stage. For each variable, the odds of the outcome for all categories compared to the odds of the outcome for a reference category were reported as the OR. Data are adjusted for age, aspirin (never vs ever), smoking (never vs ever), sex (female vs male), HTN (never vs ever), and ARBs (never vs ever).

ARB = angiotensin receptor blocker, CI = confidence interval, HTN = hypertension, OR = odds ratios.

## 4. Discussion

In this retrospective study, the association between HTN, ARB and CRC development and clinicopathological profile was investigated. Initial analysis shows a significant positive association between HTN and CRC. Nevertheless, after adjusting for confounding variables, the association became statistically insignificant. The limited sample size and the use of hospitalized controls are potential limitations that could have influenced the observed associations. Furthermore, the analysis explored the association between specific antihypertensive medications and CRC. Notably, ARB consumption among patients with HTN exhibited a potential association with an increased chance of CRC development. However, further investigation is warranted to confirm this finding. The potential association between HTN and clinicopathological characteristics of the tumors was investigated. While there was a higher percentage of poorly differentiated tumors in the patients with HTN than in the patients without HTN, there was no statistically significant difference between the 2 groups.

This study found a positive association between HTN and CRC risk that aligns with prior studies.^[19,20]^ A 2021 case-control study by Kaneko et al, utilizing data from the National Epidemiological Database of Japan, identified a weak but significant association between blood pressure and CRC risk. Their findings indicated that every 10-mm Hg increase in SBP or DBP was associated with a modest increase in the hazard ratio (HR = 1.04, 95% CI: 1.02, 1.06) for SBP and (HR = 1.06, 95% CI: 1.03, 1.09) for DBP.^[15]^ Also, Christakoudi et al (2020) conducted a prospective cohort study with over 307,000 participants from European countries. Their investigation revealed a positive association between every 10-mm Hg increase in DBP and CRC risk (HR = 1.04, 95% CI: 1.01, 1.07).^[21]^ In contrast to previous experimental studies suggesting a protective effect of ARB use on CRC risk,^[17,22]^ this study revealed a potential association between ARB use and a higher chance of CRC development among patients with HTN. According to Wang et al (2023), using ARBs may raise your risk of developing cancer in its entirety. Nevertheless, there was no discernible link between HTN drugs and CRC.^[16]^

The study of the intricate relationships between HTN and cancer has given rise to a new field called onco-hypertension. The consistent co-occurrence of cancer and HTN, alongside shared risk factors, indicates common pathophysiological mechanisms, such as inflammation and increased oxidative stress.^[23]^ Attempts to establish a causal relationship between HTN and cancer may have been hampered by shared risk factors, such as obesity, diabetes, excessive sodium intake, and low physical activity, which may affect both conditions.^[24]^

There are multiple possible biochemical pathways that could connect the use of ARBs to a higher risk of neoplasms. ARBs can influence the expression of vascular endothelial growth factor, a crucial factor in angiogenesis. Enhanced angiogenesis supports tumor growth by improving blood supply to neoplastic cells.^[25]^ ARBs also alter immune responses and inflammatory pathways. Chronic inflammation is known to be a risk factor for neoplasms because it can cause DNA damage and create a microenvironment that is favorable to tumor growth.^[26]^ Furthermore, ARBs may have an impact on the balance of reactive oxygen species, which could result in DNA damage and mutations that aid in the development of cancer. It has been demonstrated that several ARBs increase oxidative stress, which promotes carcinogenesis.^[27]^ They may also lessen the body’s capacity to stop aberrant cell growth by interfering with tumor suppressor pathways, such as the p53 pathway.^[28]^ ARBs can also alter glucose metabolism and insulin sensitivity; disruption of these metabolic pathways is linked to a higher risk of cancer.^[29]^ Also, the elevated catecholamine levels in hypertensive patients are indicative of a major role played by sympathoadrenal activity in the development of HTN. It has been shown that stimulation of adrenergic receptors contributes to the cancer progression and metastases in a variety of tumor types, including breast and colon cancer.^[30]^

Our study has several inherent limitations. The retrospective nature of the study and the case-control design restrict our ability to establish the timing of events and definitively prove cause-and-effect relationships. To partially address this limitation, we excluded cases diagnosed with HTN less than 2 years before their cancer diagnosis. Moreover, data on additional possible confounding variables that might affect the observed association, such as nutritional habits, body mass index, and amount of physical activity, were not available. Another limitation of the study is the inability to divide HTN based on SBP and DBP into 3 groups of stage 1, stage 2, and hypertensive crisis. As with other results, it is unclear if the link between HTN and cancer is causative or if biases or reverse causality could account for at least some of it. However, it is possible that the 2 conditions share pathogenesis mechanisms and risk factors.^[21]^ To determine the dose–response relationship, classification of HTN severity is recommended in future studies. It is possible to extrapolate these findings to other groups with comparable demographics and lifestyle characteristics. Consideration should be used when extending the results to populations with substantially differing exposures to the environment, genetic composition, or medical care systems.

The results of this study have consequences for public health and clinical practices regarding CRC prevention in patients with HTN or ARB users. Future investigations into the mechanisms underlying the observed associations and the setting up of personalized screening and preventative plans for people with these risk factors may be guided by these findings. CRC prevention strategies should target HTN as a more manageable risk factor, as early and routine CRC screening in hypertensive individuals may help lower the CRC development and lead to a better prognosis.

## 5. Conclusion

In conclusion, this study did not find a statistically significant association between HTN and the chance of CRC development after adjusting for confounding factors. However, our results suggest a potentially elevated risk of CRC associated with ARB consumption, which warrants further investigation. HTN has no significant association with the grade or location of tumors but is significantly associated with the stage and metastasis of tumors. This research can be considered a pilot study that provides preliminary insights into the relationship between HTN, ARB use, and CRC. However, to draw stronger conclusions, future studies should adopt a more robust design with comprehensive data collection methods and larger sample sizes.

## Acknowledgments

We extend our sincere appreciation to Rasool Akram and Firoozgar Hospitals and their dedicated staff for their invaluable support and collaboration throughout this study. We are also grateful to the Student Research Committee of the University of Iran and the Colorectal Research Center of Hazrat Rasool Akram Hospital for their support and assistance in carrying out the project.

## Author contributions

**Conceptualization:** Mohammad Rezazadeh.

**Data curation:** Amirreza Kamyabi.

**Formal analysis:** Mohammad Rezazadeh.

**Investigation:** Amirreza Kamyabi.

**Methodology:** Seidamir Pasha Tabaeian.

**Project administration:** Abolfazl Akbari.

**Resources:** Ramtin Ghamkhari Pisheh, Amirhossein Eshraghi.

**Supervision:** Shahram Agah, Abolfazl Akbari.

**Validation:** Seidamir Pasha Tabaeian.

**Visualization:** Mohammad Rezazadeh.

**Writing – original draft:** Mohammad Rezazadeh.

**Writing – review & editing:** Shahram Agah, Abolfazl Akbari.
